# Antimicrobial Susceptibility and Frequency of *bla* and *qnr* Genes in *Salmonella enterica* Isolated from Slaughtered Pigs

**DOI:** 10.3390/antibiotics10121442

**Published:** 2021-11-24

**Authors:** Alyzza Marie B. Calayag, Kenneth W. Widmer, Windell L. Rivera

**Affiliations:** 1 Pathogen-Host-Environment Interactions Research Laboratory, Institute of Biology, College of Science, University of the Philippines Diliman, Quezon City 1101, Philippines; abcalayag1@up.edu.ph; 2 International Environmental Research Institute, Gwangju Institute of Science and Technology, 123 Cheomdan-gwagiro, Buk-gu, Gwangju 61005, Korea; kennywid@gmail.com

**Keywords:** antimicrobial resistance, *bla*, extended-spectrum β-lactamase, *qnr*, *Salmonella enterica*

## Abstract

*Salmonella enterica* is known as one of the most common foodborne pathogens worldwide. While salmonellosis is usually self-limiting, severe infections may require antimicrobial therapy. However, increasing resistance of *Salmonella* to antimicrobials, particularly fluoroquinolones and cephalosporins, is of utmost concern. The present study aimed to investigate the antimicrobial susceptibility of *S. enterica* isolated from pork, the major product in Philippine livestock production. Our results show that both the *qnrS* and the *bla*_TEM_ antimicrobial resistance genes were present in 61.2% of the isolates. While *qnrA* (12.9%) and *qnrB* (39.3%) were found less frequently, co-carriage of *bla*_TEM_ and one to three *qnr* subtypes was observed in 45.5% of the isolates. Co-carriage of *bla*_TEM_ and *bla*_CTX-M_ was also observed in 3.9% of the isolates. Antimicrobial susceptibility testing revealed that the majority of isolates were non-susceptible to ampicillin and trimethoprim/sulfamethoxazole, and 13.5% of the isolates were multidrug-resistant (MDR). MDR isolates belonged to either O:3,10, O:4, or an unidentified serogroup. High numbers of *S. enterica* carrying antimicrobial resistance genes (ARG), specifically the presence of isolates co-carrying resistance to both β-lactams and fluoroquinolones, raise a concern on antimicrobial use in the Philippine hog industry and on possible transmission of ARG to other bacteria.

## 1. Introduction

*Salmonella* infections, or salmonellosis, are commonly acquired through consumption of contaminated food of animal origin. In the Philippines, *Salmonella enterica* was shown to be the leading cause of foodborne disease outbreaks from 1995–2018 [1,2]. While the disease is usually self-limiting, it may require antimicrobial therapy when the infection becomes invasive. Fluoroquinolone ciprofloxacin and extended-spectrum cephalosporin (ESC) ceftriaxone are the current treatments of choice because the emergence of antimicrobial resistance (AMR) has rendered several drugs such as ampicillin, chloramphenicol, and trimethoprim/sulfamethoxazole obsolete in salmonellosis therapy [3,4].

Resistance to β-lactams, such as ESCs, is most commonly attributed to the *bla* genes of subtypes TEM, SHV, and CTX-M, which encode for β-lactamases that hydrolyze the β-lactam ring, thereby rendering the drug inactive [5,6]. In contrast to β-lactam resistance, fluoroquinolone resistance is typically attributed to chromosomal mutations in the quinolone targets DNA gyrase and topoisomerase IV, and overexpression of efflux pumps that reduce drug accumulation [7]. However, plasmid-mediated quinolone resistance (PMQR), such as *qnr* genes, may also occur. These genes are broadly distributed worldwide and are commonly found in association with genes encoding for β-lactamases [8,9,10,11]. Consequently, *bla* and *qnr* genes have been increasingly found in bacteria isolated from livestock animals [8,9,12,13,14,15,16,17]. If motile, resistance determinants may accelerate the spread of AMR when these are taken up by non-pathogenic or pathogenic bacteria alike.

There is evidence that substantial use of antimicrobials in food-producing animals may drive the emergence of drug-resistant strains [12,18,19]. While the use of certain antimicrobials such as nitrofurans and chloramphenicol has been banned in livestock production in several parts of the world, AMR in agriculture remains a global challenge [12,19]. Monitoring AMR development in livestock and meat allows early detection of AMR emergence and prevalence [20], which can be used to design interventions to improve antimicrobial therapy and reduce resistance selection pressure [21,22]. This is generally accomplished by antimicrobial susceptibility testing (AST) and detection of antimicrobial resistance genes (ARG).

In the Philippines, pork makes up the majority of livestock production and amounts to a 3.8 M USD industry [23]. The country’s rapidly growing population is expected to further increase pork consumption and production. If left unchecked, AMR may lead to challenges in food production, food security, food safety, economic losses to the hog industry, and AMR spillover to the surrounding environment [12,18,22]. Therefore, this study aimed to investigate the antimicrobial susceptibility and frequency of β-lactamase-encoding genes (*bla*_CTX-M_, *bla*_SHV_, and *bla*_TEM_) and plasmid-mediated quinolone resistance (*qnrA*, *qnrB*, and *qnrS*) in *S. enterica* from slaughtered pigs in Metro Manila, Philippines.

## 2. Results

In total, 178 isolates were analyzed in this study. Most isolates belonged to groups O:3,10 (38.8%) and O:7 (30.3%). These were followed by groups O:4 (21.3%), and O:8 (1.7%) and O:9 (1.7%). Eleven isolates (6.2%) were not grouped and were therefore not subtyped. All isolates belonging to group O:9 carried the serovar-specific gene encoding Sdf I, indicating presumptive *S. enterica* Enteritidis (Table 1).

Results from Vitek^®^ 2 AST revealed that isolates were generally resistant to β-lactams but were susceptible to quinolones. A large number were non-susceptible to ampicillin (71.9%) and trimethoprim/sulfamethoxazole (70.8%). Non-susceptibility to key drugs, ceftazidime, ceftriaxone, and ciprofloxacin was observed in 8.4%, 7.9%, 15.7% of the isolates, respectively (Table 2). Multidrug resistance was observed in 24 (13.5%) isolates; most of which were non-susceptible to four classes of antimicrobial agents (Table 3). Although there are 15 and 14 isolates non-susceptible to the ESCs ceftazidime and ceftriaxone, respectively, Vitek^®^ 2 AST reported only one isolate with an extended-spectrum β-lactamase (ESBL)-producing phenotype.

**Figure 1:**
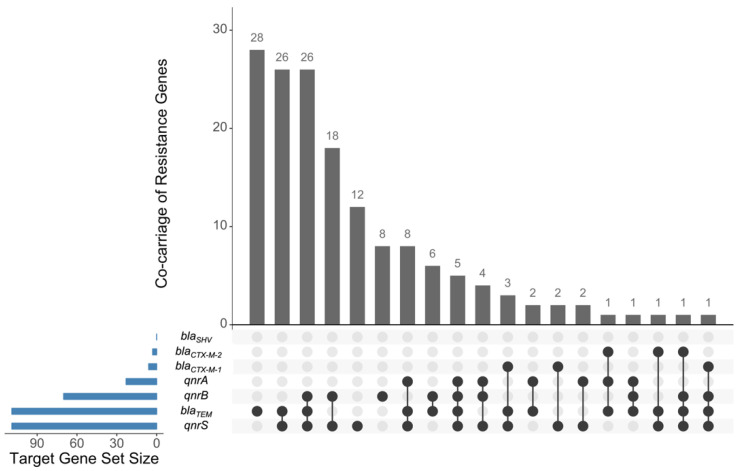
*S. enterica* isolates carrying resistance genes. Horizontal bar graphs show the total number of isolates carrying a particular antimicrobial resistance gene (ARG); vertical bar graphs show the number of isolates carrying one or more ARG. Figure was generated using UpSetR [25].

Polymerase chain reaction (PCR) assays targeting *bla* genes revealed that 61.2% and 5.1% of the isolates harbored *bla*_TEM_ and *bla*_CTX-M_ genes, respectively. No isolate carried *bla*_SHV_. CTX-M variant typing revealed that 6/9 *bla*_CTX-M_-carrying isolates carried *bla*_CTX-M-1_, and 3/9 carried *bla*_CTX-M-2_. Co-carriage of *bla*_CTX-M_ (four under the CTX-M-1 group, three under the CTX-M-2 group) and *bla*_TEM_ was observed in seven isolates. For *qnr* genes, 12.9%, 39.3%, and 61.2% were harboring the *qnrA*, *qnrB*, and *qnrS* genes, respectively. Co-carriage of *bla*_TEM_ and one to three *qnr* subtypes were found in 45.5% of the isolates (Figure 1).

## 3. Discussion

Since it has been established that ampicillin and trimethoprim/sulfamethoxazole have become obsolete in salmonellosis therapy, high non-susceptibility rates to these antimicrobials were expected. In many countries, aminopenicillins, which include ampicillin as well as trimethoprim, sulfamethoxazole, and trimethoprim/sulfonamide combinations are among the most frequently used antimicrobials in livestock production [12,26]. In the Philippines, amoxicillin, gentamicin, and trimethoprim/sulfamethoxazole are some common antimicrobials used specifically in hog farming. These antimicrobials are generally administered in all phases of pork production [26]. In this study, non-susceptibilities to ampicillin and trimethoprim/sulfamethoxazole were observed in 71.9% and 70.8% of *S. enterica*, respectively. This was similar to non-susceptibility rates reported in the Philippines in 2017, 70.5% and 80.3% to ampicillin and trimethoprim/sulfamethoxazole, respectively [27]. While non-susceptibility to the latter appeared to be common with all serogroups tested in the present study, non-susceptibility to ampicillin was not observed in the O:9 group. Phongaran et al. [13] reported that 69.0% of *Salmonella* isolated from hogs in Thailand were resistant to ampicillin. However, in this study, only 35.7% were resistant to trimethoprim/sulfamethoxazole. One study conducted among pork in Vietnam reported that 36.7% of *Salmonella* isolates were resistant to trimethoprim/sulfamethoxazole and only 41.3% to ampicillin [28]. Conversely, low rates of resistance (<5%) to ESC and ciprofloxacin were reported in both studies [13,28], while the present study reported rates that were almost twice as high (<10%) and that were mainly due to isolates from groups O:3,10 and O:4. However, only one of the isolates in this study, which belonged to the O:3,10 group, produced an ESBL phenotype. Non-susceptibility of other isolates to ESC must be due to broad-spectrum β-lactamases that are not affected by β-lactamase inhibitors. Three isolates of unidentified serogroups were non-susceptible to carbapenems, and one which showed the MDR pattern Pen, Pen/IB, Car, Flu. Carbapenems are not used in agriculture in Southeast Asia [12]; thus, this emerging non-susceptibility to carbapenems may warrant further investigation.

In this study, multidrug resistance was observed in 13.5% of *S. enterica* isolates from O:3,10 (11), O:4 (9), and unidentified serogroups (3). Reports of multidrug-resistant (MDR) *Salmonella* isolated from hogs in other Southeast Asian countries are higher (30–40%) [13,28]. In particular, multidrug resistance in the present study was five times lower than that reported in the Philippines in 2017 [27]. In other countries, higher rates (70–80%) of MDR *Salmonella* isolated from pork and the pork production chain were observed [15,29]. Of the 24 MDR *S. enterica* isolates in the present study, 15 and 8 were non-susceptible to ESC and fluoroquinolones, respectively, the current drug options in treating salmonellosis. Multidrug resistance is a challenge, as it narrows down the options for antimicrobial therapy.

The majority of studies on *bla* genes and livestock animals in Southeast Asian countries are focused on *E. coli* in which *bla*_TEM_ and *bla*_CTX-M_ are the most frequently identified *bla* genes [12]. In *Salmonella*, *bla*_TEM_ appears to be the most common. Lalruatdiki et al. [14] observed that in India, 30% of *Salmonella* isolated from a pig population were carrying *bla*_TEM_, and 10% carried *bla*_CTX-M_. Similarly, a survey in Italy observed approximately 21% of *Salmonella* recovered from pork samples had the presence of *bla*_TEM_ [30]. Co-carriage of *bla*_CTX-M_ and *bla*_TEM_ has also been observed in ESBL-producing *Salmonella* from pigs [14,31]. In the present study, co-carriage of *bla*_TEM_ and *bla*_CTX-M_ was found in seven (3.9%) isolates. However, none of these isolates were ESBL-producing strains which could suggest that they carry silent copies of *bla* genes. The presence of inactive ESBL genes has been reported in *Klebsiella pneumoniae* [32] and in *Escherichia coli* [33]. The only ESBL-producing *Salmonella* in this study was carrying only *bla*_TEM_. It is possible that this *bla*_TEM_ subtype could encode ESBLs, or the isolate could be carrying other ESBL-encoding genes. Among isolates carrying *bla*_TEM_, 93.6% (102/109) were non-susceptible to ampicillin, but susceptible to ampicillin/clavulanic acid and piperacillin/tazobactam. Conversely, among isolates non-susceptible to ampicillin, 20.3% (26/128) were not carrying *bla*_TEM_. Furthermore, some of these isolates exhibited non-susceptibility to ampicillin/clavulanic acid and ceftriaxone. While most *bla*_TEM_ in the study possibly confer only broad-spectrum β-lactam resistance considering the high rates of non-susceptibility to ampicillin, its presence in combination with other resistance determinants could render an isolate multidrug-resistant.

We report in this study that 71.3% of *S. enterica* isolates harbored PMQR. The genes *qnrA*, *qnrB*, and *qnrS* were observed in 12.9%, 39.3%, and 61.2% of the isolates, respectively. While Qnr proteins offer only low resistance against quinolones, these have been shown to broaden the mutant selection window in bacteria [7]. Lin et al. [16] demonstrated that ciprofloxacin resistance conferred by PMQR is even comparable to that of quinolone target mutations. However, Temmerman et al. [34] reported a limited role of PMQR in quinolone resistance. Nevertheless, investigating PMQR is significant because it often carries other ARG [9,10,11]. Prevalence rates of *qnr* genes appear to vary among samples and geographical locations. Cameron-Veas et al. [15] reported that 15% of *S. enterica* isolated from a pork production chain in Brazil were carrying *qnrB*, and none of them carried *qnrA* and *qnrS*. A separate study in China reported the prevalence of *qnrD* (3%), *qnrB* (16%), and *qnrS* (66%) [16] in foodborne *Salmonella*. In Thailand and in Laos, Sinwat et al. [17] found only 1–8% of *S. enterica* isolated from pork carried the same *qnr* genes. This highlights the importance of a national surveillance of ARG since it appears that individual countries seem to have different prevalence rates.

Several studies have also reported the association of *qnr* genes with *bla* genes. One MDR *Salmonella* isolated from a piglet in Spain carried both *qnrB* and *bla*_CTX-M_. Moawad et al. [8] found that 33% of *Salmonella* from poultry and beef in Egypt were carrying *qnr* genes and either *bla*_CTX-M_, *bla*_TEM_, or both. Whether *qnr* and *bla* genes reside within the same plasmid was not confirmed in either of the studies. However, Penha Filho et al. [9] recently isolated *Salmonella* from poultry in Brazil which carried both *bla*_CTX-M-2_ and *qnrB* in the same plasmid. In clinical isolates of *S. enterica*, *Escherichia coli*, and *Klebsiella pneumoniae*, *qnr* genes have also been found within the same plasmid as that of *bla*_TEM_ or *bla*_CTX-M_ [10,11]. In the present study, 81 *bla*_TEM_-carrying isolates and all 9 *bla*_CTX-M_-carrying isolates were harboring one to three *qnr* subtypes.

The increasing prevalence of MDR *Salmonella* in livestock animals has been widely reported [13,15,27,29] and is mainly attributed to the inappropriate use of antimicrobial agents in veterinary medicine [18,19]. MDR *Salmonella* in pork may potentially have an even larger impact on public health due to discharge of livestock waste into water sources. A recent survey in Ho Chi Minh City of surface water and produce indicated 17.5% of vegetable samples were positive for *Salmonella*, and of all isolates recovered, 26.5% were considered MDR *Salmonella*. The authors suggested that livestock runoff into surface water used for irrigation and agriculture processing were likely contamination sources [35]. We report that 89.4% of *S. enterica* isolated from slaughtered pork were non-susceptible to at least one antimicrobial agent and 13.5% were MDR. All MDR isolates belonged to either O:3,10, O:4, or a different serogroup that was not tested. Majority of the isolates were also harboring *bla*_TEM_ that possibly encode broad-spectrum β-lactamases, and *qnrS*, which could facilitate emergence of mutations that target quinolone resistance.

While worldwide AMR surveillance has allowed the determination of the evolution of resistance, national surveillance will allow countries to create policies that would fit their needs. Generation of local information on AMR and antimicrobial consumption in the veterinary and agricultural sectors will allow the development of relevant approaches to tackle AMR [21,22]. This is highly important for low- and middle-income countries (LMICs) as strategies proven effective to work in developed countries may not be suitable for LMICs. Attention to AMR in the agricultural sector began in the Philippines only recently, and further surveillance is necessary to identify emerging resistant *S. enterica* in the pork production chain.

## 4. Materials and Methods

### 4.1. Sample Collection

The study population consisted of freshly slaughtered hogs from seven abattoirs across four districts of Metro Manila, Philippines. The abattoirs selected were all registered with the National Meat Inspection Service (NMIS) of the Philippines and were the major slaughtering facilities in each district. Informed consent was obtained from the NMIS; hence, ethics approval was waived for this particular study. Animal slaughter and evisceration were performed according to national regulations. Informed consent was also obtained from veterinarians in charge of the abattoirs, and farm owners for sample collection. Tissue samples from hog tonsils and jejunum were collected post-slaughter and under the supervision of a veterinarian. Sample collection was performed as previously described [27]. Briefly, tissues were collected from each hog upon evisceration using sterile forceps and scissors, and then immediately transferred into sterile bags. All samples were kept chilled upon collection and during transport and were immediately processed in the laboratory.

### 4.2. Bacterial Isolation and Identification

Bacterial isolates analyzed in this study included isolates from a previous study that were not tested for antimicrobial susceptibility, collected from June to December 2013 (*n* = 117) [27], with isolates collected from June to December 2014 (*n* = 61). Bacteria were first enriched prior to isolation as previously described [27]. Briefly, 25 g of each sample was transferred to 225 mL buffered peptone water (BPW) and incubated overnight at 35 °C. Afterward, 100 µL of pre-enriched bacterial culture in BPW was inoculated into 10 mL Rappaport-Vassiliadis broth (RVB), and then incubated overnight at 42 °C for selective enrichment of *S. enterica*. RVB cultures were inoculated onto brilliant green agar (BGA) and xylose lysine deoxycholate agar (XLD) and then incubated overnight at 35 °C for isolation. Presumptive *S. enterica* were then inoculated onto nutrient agar (NA) and incubated overnight at 35 °C for subsequent total DNA extraction.

Total DNA was extracted by harvesting colonies using a sterile 1 μL loop and suspending these in 100 μL TE buffer (10 mM Tris, 1 mM EDTA at pH 8.0). The suspension was boiled for 10 min, and pelleted at 6000 rpm for 5 min. The supernatant was collected and then stored at −20 °C until use. These DNA extracts were used in both PCR-based identification of *S. enterica* and detection of ARG.

Each PCR reaction for *S. enterica* identification contained 2 μL DNA, 10 pmol each of forward and reverse primers, and HiPi PCR Premix (Elpis Biotech, Daejeon, Korea) in a final volume of 20 μL. Amplification of a 244 bp region in the species-specific *invA* gene was performed as previously described [36]. PCR products were subsequently analyzed via capillary electrophoresis. *S. enterica* KCTC 2421 was used as a positive control.

### 4.3. Molecular Characterization of S. enterica

All isolates were subjected to a two-step PCR assay to characterize the somatic and flagellar antigens. The first step included 12 primers to identify the most common *Salmonella* serogroups: O:2, O:4, O:6,7, O:8, O:9, and O:3,10 and was based on a previously described protocol [37]. The second step included nine primers to identify the first-phase flagellar antigens (H_1_) and two to identify a fragment of the gene encoding the *Salmonella* difference fragment I (Sdf I) unique to *S. enterica* Enteritidis. The primers used are listed in Table 4.

Each reaction in the second step contained 2 μL DNA, 10 pmol each of forward and reverse primers, and 6.25 μL GoTaq^®^ Green Master Mix (Promega, Madison, USA) in a final volume of 12.5 μL. For Sdf I, PCR was carried out under the following conditions: initial denaturation step at 95 °C for 3 min; 30 cycles of denaturation at 95 °C for 30 s, annealing at 58 °C for 30 s, extension at 72 °C for 1 min; and a final extension step at 72 °C for 5 min. For the H_1_ antigens, including G complex alleles, monoplex PCR for each target gene was carried out under the following conditions: initial denaturation step at 95 °C for 2 min; 35 cycles of denaturation at 95 °C for 30 s, annealing at 55 °C for 30 s, extension at 72 °C for 30 s; and a final extension step at 72 °C for 5 min.

Amplicons were analyzed in 1.5% (monoplex PCR products) or 2% (multiplex PCR products) agarose gels stained either with GelRed™ Nucleic Acid Gel Stain (Biotium, Fremont, USA) or SYBR^®^ Safe DNA Gel Stain (Invitrogen, Carlsbad, USA) (1:10,000). Amplicons were allowed to separate at 100 V for 20–30 min and then viewed in a gel documentation system. KAPA™ Universal Ladder (KAPA Biosystems, Boston, USA) was used to estimate the molecular weights of the products.

### 4.4. Bacterial Storage and Recovery

*Salmonella enterica* isolates were maintained as glycerol stocks until further analyses. Glycerol stocks were prepared by gently mixing 300 μL of sterile 80% glycerol solution to 700 μL of overnight culture of *S. enterica* in tryptic soy broth (TSB) and then stored at −20 °C.

To recover glycerol stocks, 200 μL of the culture was inoculated into 800 μL TSB and then incubated overnight at 35 °C. TSB cultures were then inoculated onto XLD and incubated overnight at 35 °C to ensure purity of the culture. Typical *Salmonella* colonies were maintained in NA until subsequent antimicrobial susceptibility testing.

### 4.5. Antimicrobial Susceptibility Testing

The Vitek^®^ 2 AST system was used to generate antimicrobial susceptibility profiles of the isolates. It automatically classifies isolates into susceptible, intermediate, or resistant to a particular antimicrobial agent based on the latest breakpoints provided by the Clinical and Laboratory Standards Institute (CLSI). Multidrug resistance was defined as non-susceptibility to at least one antimicrobial agent in three or more antimicrobial categories as recommended by Magiorakos et al. [24].

Inoculum preparation for the automated AST was followed as previously described [27]. Vitek^®^ 2 AST-N261 cards were used which contain 15 antimicrobials including amikacin, amoxicillin/clavulanate, ampicillin, cefepime, cefoxitin, ceftazidime, ceftriaxone, ciprofloxacin, colistin, ertapenem, gentamicin, imipenem, meropenem, piperacillin/tazobactam, and trimethoprim/sulfamethoxazole and an ESBL test. Colistin was not tested because there are currently no CLSI breakpoints available for *Salmonella* spp. The ESBL test included cefepime, ceftriaxone, and ceftazidime alone and in combination with clavulanic acid. Vitek^®^ 2 AST reports either a positive or negative ESBL test. Each test run was accompanied with AST for *E. coli* ATCC 25922 (negative control for ESBL test) and *K. pneumoniae* ATCC 600703 (positive control for ESBL test) (Supplementary Table S1).

### 4.6. Detection of bla and qnr Genes

*S. enterica* isolates were screened for β-lactamase-encoding genes (*bla*_CTX-M_, *bla*_SHV_, and *bla*_TEM_) and quinolone resistance genes (*qnrA*, *qnrB*, and *qnrS*) using monoplex PCR assays. The primers used are listed in Table 5. For *bla* genes, each reaction contained 2 μL DNA, 10 pmol each of forward and reverse primers, and AccuPower^®^ PCR Premix (Bioneer, Daejeon, Korea) or Maxime PCR Premix (*i*-StarTaq™ GH) (iNtRON Biotechnology, Seongnam, Korea) in a final volume of 20 μL. PCR was carried out under the following conditions: initial denaturation step at 95 °C for 3 min; 30 cycles of denaturation at 95 °C for 30 s, annealing at 58 °C for *bla*_CTX-M_, 56 °C for *bla*_SHV_, and 50 °C for *bla*_TEM_ for 30 s, extension at 72 °C for 1 min; and a final extension step at 72 °C for 10 min. For *qnr* genes, each reaction contained 2 μL DNA, 10 pmol each of forward and reverse primers, and 6.25 μL GoTaq^®^ Green Master Mix in a final volume of 12.5 μL. PCR was carried out under the following conditions: initial denaturation step at 95 °C for 5 min; 33 cycles of denaturation at 95 °C for 1 min, annealing at 60 °C for 1 min, extension at 72 °C for 1 min; and a final extension step at 72 °C for 10 min.

*S. enterica* isolates carrying *bla*_CTX-M_ were subjected to further PCR assays to identify CTX-M variants. The primers used in CTX-M variant typing are listed in Table 3. Each reaction contained 2 μL DNA, 10 pmol each of forward and reverse primers, and 6.25 μL GoTaq^®^ Green Master Mix in a final volume of 12.5 μL. Amplification was performed as previously described [6]. Amplicons were analyzed as described above.

## Figures and Tables

**Table 1:** Molecular characterization of *S. enterica* isolates.

Serogroup	First-Phase Flagellar (H_1_) Antigens					Sdf I	Other
	H:d	H: e,h	H:g	H:i	H:r		
O:3,10 (*n* = 69)		39	23				7
O:4 (*n* = 38)	1	2		25	1		9
O:7 (*n* = 54)		2	27	9	10		6
O:8 (*n* = 3)				2			1
O:9 (*n* = 3)						3	
Other (*n* = 11)							

No isolates under group O:2 were detected.

**Table 2:** Non-susceptibility levels of 178 *S. enterica* isolates against different antimicrobial agents.

Class	Antimicrobial	% Non-Susceptibility
Penicillin	Ampicillin	71.9% (±8.8)
Penicillin/β-lactamase inhibitor	Amoxicillin/clavulanic acid	10.1% (±5.9)
Antipseudomonal penicillin/β-lactamase inhibitor	Piperacillin/tazobactam	0.6% (±1.5)
Extended-spectrum cephalosporin	Ceftazidime	8.4% (+5.4)
	Ceftriaxone	7.9% (±5.3)
	Cefepime	0.0% (±0)
Carbapenem	Ertapenem	0.0% (±0)
	Imipenem	1.7% (±2.5)
	Meropenem	0.0% (±0)
Fluoroquinolone	Ciprofloxacin	15.7% (±7.1)
Folate pathway inhibitor	Trimethoprim/sulfamethoxazole	70.8% (±8.9)

Antimicrobials classified into categories based on recommendations of Magiorakos et al. [24]. Non-susceptibility to non-ESCs, cephamycins, and aminoglycosides are not shown as these antimicrobial agents are not clinically effective, although they may appear active in vitro. Values in parenthesis are ± 95% binomial confidence intervals.

**Table 3:** Multidrug resistance patterns of 24 *S. enterica* isolates.

Number of *S. enterica* Isolates	Multidrug Resistance Pattern ^1^
1	Pen, Pen/BI, APen/BI, ESC, Flu
14	Pen, Pen/BI, FPI, ESC
1	Pen, Pen/BI, FPI, Car
1	Pen, Pen/BI, Car, Flu
7	Pen, FPI, Flu

^1^ Pen, penicillin; Pen/BI, penicillin/β-lactamase inhibitor; APen/BI; antipseudomonal penicillin/β-lactamase inhibitor; ESC, extended-spectrum cephalosporin; Flu, fluoroquinolone; FPI, folate pathway inhibitor; Car, carbapenem.

**Table 4:** Targets and primers used in molecular characterization of *S. enterica*.

Target	Nucleotide Sequence (5′-3′)	Amplicon Length (bp)	Reference
O:4	F: GGCTTCCGGCTTTATTGG R: TCTCTTATCTGTTCGCCTGTTG	561	[38]
O:9	F: GAGGAAGGGAAATGAAGCTTTT R: TAGCAAACTGTCTCCCACCATAC	615	[39]
O:2, O:9	F: CTTGCTATGGAAGACATAACGAACC R: CGTCTCCATCAAAAGCTCCATAGA	258	[39]
O:6,7	F: ATTTGCCCAGTTCGGTTTG R: CCATAACCGACTTCCATTTCC	341	[38]
O:8	F: CGTCCTATAACCGAGCCAAC R: R: CTGCTTTATCCCTCTCACCG	397	[38]
O:3, 10	F: GATAGCAACGTTCGGAAATTC R: CCCAATAGCAATAAACCAAGC	281	[38]
Sense60	F: GCAGATCAACTCTCAGACCCTGGG		[40]
H:r	R: AAGTGACTTTTCCATCGGCTG	275	[41]
H:i	R: ATAGCCATCTTTACCAGTTCC	250	[41]
H:e,h	R: AACGAAAGCGTAGCAGACAAG	200	[41]
H:b	R: CGCACCAGTCYWACCTAAGGCGG	150	[41]
H:d	F: CCCGAAAGAAACTGCTGTAACCG R: TGGATATCAGTATTGCTCTGGGC	100	[41]
G complex alleles (H:g)	F: GTGATCTGAAATCCAGCTTCAAG R: AAGTTTCGCACTCTCGTTTTTGG	500	[41]
Sdf I	F: TGTGTTTTATCTGATGCAAGAGG R: CGTTCTTCTGGTACTTACGATGAC	333	[42]

**Table 5:** Resistance gene targets and primers used in the present study.

Target Gene	Nucleotide Sequence (5′-3′)	Amplicon Length (bp)	Reference
*bla*_SHV_	F: ATGCGTTATATTCGCCTGTG	747	[43]
	R: TGCTTTGTTATTCGGGCCAA		
*bla*_TEM_	F: TCGCCGCATACACTATTCTCAGAAT GA	445	[5]
	R: ACGCTCACCGGCTCCAGATTTAT		
*bla*_CTX-M_	F: ATGTGCAGYACCAGTAARGTKATGG C	593	[44]
	R: TGGGTRAARTARGTSACCAGAAYCA GCGG		
*bla*_CTX-M-1_	F: AAAAATCACTGCGCCAGTTC	415	[45]
	R: AGCTTATTCATCGCCACGTT		
*bla*_CTX-M-2_	F: CGATATCGTTGGTGGTRCCAT	404	[6]
	R: CGTTAACGGCACGATGAC		
*bla*_CTX-M-9_	F: CAAAGAGAGTGCAACGGATG	205	[45]
	R: ATTGGAAAGCGTTCATCACC		
*bla*_CTX-M-8/25_	F: AACCCACGATGTGGGTAGC		[45]
*bla*_CTX-M-8_	R: TCGCGTTAAGCGGATGATGC	666	
*bla*_CTX-M-25_	R: GCACGATGACATTCGGG	327	
*qnrA*	F: AGAGGATTTCTCACGCCAGG	580	[46]
	R: TGCCAGGCACAGATCTTGAC		
*qnrB*	F: GGAATAGAAATTCGCCACTG	264	[47]
	R: TTTGCTGTTCGCCAGTCGAA		
*qnrS*	F: GCAAGTTCATTGAACAGGGT	428	[46]
	R: TCTAAACCGTCGAGTTCGGCG		

## Data Availability

The data presented in this study are available in Supplementary Material.

## Supplementary Materials

The following are available online at https://www.mdpi.com/article/10.3390/antibiotics10121442/s1. Table S1: Minimum inhibitory concentrations of antimicrobials as determined by Vitek^®^ 2 antimicrobial susceptibility testing. Table S2: Distribution of *S. enterica* into serogroups and H_1_/Sdf I typing.
