# Supplementary material for: Antimicrobial Susceptibility and Frequency of bla and qnr Genes in Salmonella enterica Isolated from Slaughtered Pigs
Source: Antibiotics (Basel). 2021 Nov 24;10(12):1442. doi: 10.3390/antibiotics10121442 (PMC8698178; doi:10.3390/antibiotics10121442)
Supplement: Supplementary file 1 [file antibiotics-10-01442-s001.zip › TableS1_revised.pdf]

Table S1. Minimum inhibitory concentrations of antimicrobials as determined by Vitek® 2 antimicrobial susceptibility testing.

Abattoir	Isolate	Ampicillin	Amoxicillin /Clavulanic acid	Piperacillin/Tazo-bactam	Ceftazidime	Ceftriaxone	Cefepime	Ertapenem	Imipenem	Meropenem	Ciprofloxacin	Trimethoprim/Sul-famethoxazole
	AC-003	≥32	8/4	≤4/4	≤1	≤1	≤1	≤0.5	≤0.25	≤0.25	≤0.25	≥320
	AC-004	≥32	4/2	≤4/4	≤1	≤1	≤1	≤0.5	≤0.25	≤0.25	≤0.25	≥320
	AC-005	≥32	4/2	≤4/4	≤1	≤1	≤1	≤0.5	≤0.25	≤0.25	≤0.25	≥320
	AC-006	≥32	4/2	≤4/4	≤1	≤1	≤1	≤0.5	≤0.25	≤0.25	≤0.25	≥320
	AC-007	≥32	4/2	≤4/4	≤1	≤1	≤1	≤0.5	≤0.25	≤0.25	≤0.25	≥320
	AC-008	≥32	4/2	≤4/4	≤1	≤1	≤1	≤0.5	≤0.25	≤0.25	≤0.25	≥320
	AC-009	≤2	2/1	≤4/4	≤1	≤1	≤1	≤0.5	≤0.25	≤0.25	≥4	≤20
	AC-010	≥32	4/2	≤4/4	≤1	≤1	≤1	≤0.5	≤0.25	≤0.25	≤0.25	≥320
	AC-253	≥32	4/2	≤4/4	≤1	≤1	≤1	≤0.5	≤0.25	≤0.25	≤0.25	≥320
	AC-254	≥32	4/2	≤4/4	≤1	≤1	≤1	≤0.5	≤0.25	≤0.25	≤0.25	≥320
	AC-255	≥32	4/2	≤4/4	≤1	≤1	≤1	≤0.5	≤0.25	≤0.25	≤0.25	≥320
	AC-256	≥32	4/2	≤4/4	≤1	≤1	≤1	≤0.5	≤0.25	≤0.25	≤0.25	≤20
	AC-257	≥32	4/2	≤4/4	≤1	≤1	≤1	≤0.5	≤0.25	≤0.25	≤0.25	≥320
	AC-258	≥32	4/2	≤4/4	≤1	≤1	≤1	≤0.5	≤0.25	≤0.25	≤0.25	≥320
A	AC-259	≥32	4/2	≤4/4	≤1	≤1	≤1	≤0.5	≤0.25	≤0.25	≤0.25	≥320
	AC-260	≥32	4/2	≤4/4	≤1	≤1	≤1	≤0.5	≤0.25	≤0.25	≤0.25	≥320
	AC-261	≥32	4/2	≤4/4	≤1	≤1	≤1	≤0.5	≤0.25	≤0.25	≤0.25	≥320
	AC-262	≥32	4/2	≤4/4	≤1	≤1	≤1	≤0.5	≤0.25	≤0.25	≤0.25	≥320
	AC-263	≥32	4/2	≤4/4	≤1	≤1	≤1	≤0.5	≤0.25	≤0.25	≤0.25	≥320
	AC-264	≥32	4/2	≤4/4	≤1	≤1	≤1	≤0.5	≤0.25	≤0.25	≤0.25	≥320
	AC-265	≥32	4/2	≤4/4	≤1	≤1	≤1	≤0.5	≤0.25	≤0.25	≤0.25	≥320
	AC-267	≥32	4/2	≤4/4	≤1	≤1	≤1	≤0.5	≤0.25	≤0.25	≤0.25	≥320
	AC-268	≥32	4/2	≤4/4	≤1	≤1	≤1	≤0.5	≤0.25	≤0.25	≤0.25	≥320
	AC-270	≥32	4/2	≤4/4	≤1	≤1	≤1	≤0.5	≤0.25	≤0.25	≤0.25	≥320
	AC-272	≥32	4/2	≤4/4	≤1	≤1	≤1	≤0.5	≤0.25	≤0.25	≤0.25	≥320
	AC-273	≥32	4/2	≤4/4	≤1	≤1	≤1	≤0.5	≤0.25	≤0.25	≤0.25	≥320
	AC-274	≥32	4/2	≤4/4	≤1	≤1	≤1	≤0.5	≤0.25	≤0.25	≤0.25	≤20
	AC-275	≥32	4/2	≤4/4	≤1	≤1	≤1	≤0.5	≤0.25	≤0.25	≤0.25	≥320

	AC-276	≥32	4/2	≤4/4	≤1	≤1	≤1	≤0.5	≤0.25	≤0.25	≤0.25	≥320
	AM-102	≤2	2/1	≤4/4	≤1	≤1	≤1	≤0.5	≤0.25	≤0.25	≤0.25	≥320
	AM-105	≥32	8/4	≤4/4	≤1	≤1	≤1	≤0.5	≤0.25	≤0.25	≤0.25	≥320
	AM-106	≤2	2/1	≤4/4	≤1	≤1	≤1	≤0.5	≤0.25	≤0.25	≤0.25	≥320
	AM-108	≥32	8/4	≤4/4	≤1	≤1	≤1	≤0.5	≤0.25	≤0.25	≤0.25	≥320
	AM-109	≤2	2/1	≤4/4	≤1	≤1	≤1	≤0.5	≤0.25	≤0.25	≤0.25	≥320
	AM-112	≥32	8/4	≤4/4	≤1	≤1	≤1	≤0.5	≤0.25	≤0.25	≤0.25	≥320
	AM-113	≥32	4/2	≤4/4	≤1	≤1	≤1	≤0.5	≤0.25	≤0.25	≤0.25	≥320
	AM-114	≤2	2/1	≤4/4	≤1	≤1	≤1	≤0.5	≤0.25	≤0.25	≤0.25	≤20
	AM-115	≥32	4/2	≤4/4	≤1	≤1	≤1	≤0.5	≤0.25	≤0.25	≤0.25	≥320
	AM-116	≤2	2/1	≤4/4	≤1	≤1	≤1	≤0.5	≤0.25	≤0.25	≤0.25	≥320
	AM-118	≤2	2/1	≤4/4	≤1	≤1	≤1	≤0.5	≤0.25	≤0.25	≤0.25	≥320
B	AM-119	≤2	2/1	≤4/4	≤1	≤1	≤1	≤0.5	≤0.25	≤0.25	≤0.25	≤20
	AM-120	≤2	2/1	≤4/4	≤1	≤1	≤1	≤0.5	≤0.25	≤0.25	≤0.25	≤20
	AM-121	≤2	2/1	≤4/4	≤1	≤1	≤1	≤0.5	≤0.25	≤0.25	≤0.25	≤20
	NS-191	≤2	2/1	≤4/4	≤1	≤1	≤1	≤0.5	≤0.25	≤0.25	≤0.25	≥320
	NS-192	≥32	4/2	≤4/4	≤1	≤1	≤1	≤0.5	≤0.25	≤0.25	≤0.25	≥320
	NS-193	≤2	2/1	≤4/4	≤1	≤1	≤1	≤0.5	≤0.25	≤0.25	≤0.25	≤20
	AM-247	≤2	2/1	≤4/4	≤1	≤1	≤1	≤0.5	≤0.25	≤0.25	≤0.25	≤20
	AM-248	≤2	2/1	≤4/4	≤1	≤1	≤1	≤0.5	≤0.25	≤0.25	≤0.25	≥320
	AM-249	≤2	2/1	≤4/4	≤1	≤1	≤1	≤0.5	≤0.25	≤0.25	≤0.25	≥320
	AM-250	≤2	2/1	≤4/4	≤1	≤1	≤1	≤0.5	≤0.25	≤0.25	≤0.25	≥320
	AM-251	≤2	2/1	≤4/4	≤1	≤1	≤1	≤0.5	≤0.25	≤0.25	≤0.25	≥320
	AM-252	≤2	2/1	≤4/4	≤1	≤1	≤1	≤0.5	≤0.25	≤0.25	≤0.25	≥320
C	AT-098	≤2	2/1	≤4/4	≤1	≤1	≤1	≤0.5	≤0.25	≤0.25	≤0.25	≤20
	AT-099	≤2	2/1	≤4/4	≤1	≤1	≤1	≤0.5	≤0.25	≤0.25	≤0.25	≤20
	AV-066	≤2	2/1	≤4/4	≤1	≤1	≤1	≤0.5	≤0.25	≤0.25	≤0.25	≤20
	AV-067	≤2	2/1	≤4/4	≤1	≤1	≤1	≤0.5	≤0.25	≤0.25	≤0.25	≤20
D	AV-068	≤2	2/1	≤4/4	≤1	≤1	≤1	≤0.5	≤0.25	≤0.25	0.5	≥320
	AV-070	≤2	2/1	≤4/4	≤1	≤1	≤1	≤0.5	≤0.25	≤0.25	0.5	≥320
	AV-072*	≥32	≥32/16	≤4/4	≥16	4	≤1	≤0.5	0.5	≤0.25	≤0.25	≥320
	NK-011	≥32	4/2	8/4	≤1	≤1	≤1	≤0.5	≤0.25	≤0.25	0.5	≤20

	NK-012	≥32	4/2	8/4	≤1	≤1	≤1	≤0.5	≤0.25	≤0.25	0.5	≤20
	NK-013	≤2	2/1	≤4/4	≤1	≤1	≤1	≤0.5	≤0.25	≤0.25	≤0.25	≥320
	NK-015	≥32	4/2	8/4	≤1	≤1	≤1	≤0.5	≤0.25	≤0.25	0.5	≤20
	NK-016	≤2	2/1	≤4/4	≤1	≤1	≤1	≤0.5	≤0.25	≤0.25	≤0.25	≥320
	NK-017	≤2	2/1	≤4/4	≤1	≤1	≤1	≤0.5	≤0.25	≤0.25	≤0.25	≥320
	NK-018*	≥32	≥32/16	≤4/4	≥16	4	≤1	≤0.5	≤0.25	≤0.25	≤0.25	≥320
	NK-019	≤2	2/1	≤4/4	≤1	≤1	≤1	≤0.5	≤0.25	≤0.25	≤0.25	≤20
	NK-021*	≥32	4/2	8/4	≤1	≤1	≤1	≤0.5	≤0.25	≤0.25	0.5	≥320
	NK-022*	≥32	4/2	8/4	≤1	≤1	≤1	≤0.5	≤0.25	≤0.25	0.5	≥320
	NK-023	≤2	2/1	≤4/4	≤1	≤1	≤1	≤0.5	≤0.25	≤0.25	≤0.25	≥320
	NK-024	≥32	4/2	8/4	≤1	≤1	≤1	≤0.5	≤0.25	≤0.25	0.5	40
	NK-025	≥32	4/2	8/4	≤1	≤1	≤1	≤0.5	≤0.25	≤0.25	0.5	≤20
	NK-026	≥32	4/2	8/4	≤1	≤1	≤1	≤0.5	≤0.25	≤0.25	0.5	≤20
	NK-027	≤2	2/1	≤4/4	≤1	≤1	≤1	≤0.5	≤0.25	≤0.25	≤0.25	≥320
	NK-029	≤2	2/1	≤4/4	≤1	≤1	≤1	≤0.5	≤0.25	≤0.25	≤0.25	≥320
	NK-031	≥32	4/2	≤4/4	≤1	≤1	≤1	≤0.5	≤0.25	≤0.25	≤0.25	≤20
	NK-032*	≥32	4/2	≤4/4	≤1	≤1	≤1	≤0.5	≤0.25	≤0.25	≥4	≥320
	NK-033	≤2	2/1	≤4/4	≤1	≤1	≤1	≤0.5	≤0.25	≤0.25	≤0.25	≥320
	NK-035	≥32	4/2	≤4/4	≤1	≤1	≤1	≤0.5	≤0.25	≤0.25	0.5	≤20
	NK-036	≥32	4/2	8/4	≤1	≤1	≤1	≤0.5	≤0.25	≤0.25	0.5	≤20
E	NK-037	≥32	4/2	8/4	≤1	≤1	≤1	≤0.5	≤0.25	≤0.25	0.5	≤20
	NK-093	≤2	2/1	≤4/4	≤1	≤1	≤1	≤0.5	≤0.25	≤0.25	≤0.25	≤20
	NK-094	≤2	2/1	≤4/4	≤1	≤1	≤1	≤0.5	≤0.25	≤0.25	≤0.25	≤20
	NK-194	≤2	2/1	≤4/4	≤1	≤1	≤1	≤0.5	≤0.25	≤0.25	≤0.25	≥320
	NK-195	≤2	2/1	≤4/4	≤1	≤1	≤1	≤0.5	≤0.25	≤0.25	≤0.25	≥320
	NK-196	≤2	2/1	≤4/4	≤1	≤1	≤1	≤0.5	≤0.25	≤0.25	≤0.25	≥320
	NK-197	≤2	2/1	≤4/4	≤1	≤1	≤1	≤0.5	≤0.25	≤0.25	≤0.25	≥320
	NK-198	≤2	2/1	≤4/4	≤1	≤1	≤1	≤0.5	≤0.25	≤0.25	≤0.25	≥320
	NK-199	≥32	4/2	8/4	≤1	≤1	≤1	≤0.5	≤0.25	≤0.25	2	≤20
	NK-200	≥32	4/2	≤4/4	≤1	≤1	≤1	≤0.5	≤0.25	≤0.25	≤0.25	≥320
	NK-201	≥32	4/2	≤4/4	≤1	≤1	≤1	≤0.5	≤0.25	≤0.25	≤0.25	≥320
	NK-202	≥32	4/2	8/4	≤1	≤1	≤1	≤0.5	≤0.25	≤0.25	2	≤20

	NK-203	≥32	8/4	8/4	≤1	≤1	≤1	≤0.5	≤0.25	≤0.25	2	≤20
	NK-204*	≥32	4/2	8/4	≤1	≤1	≤1	≤0.5	≤0.25	≤0.25	2	60
	NK-205	≥32	4/2	8/4	≤1	≤1	≤1	≤0.5	≤0.25	≤0.25	2	≤20
	NK-206*	≥32	4/2	8/4	≤1	≤1	≤1	≤0.5	≤0.25	≤0.25	2	60
	NK-208	≥32	4/2	8/4	≤1	≤1	≤1	≤0.5	≤0.25	≤0.25	2	≤20
	NK-341	≥32	4/2	8/4	≤1	≤1	≤1	≤0.5	≤0.25	≤0.25	2	≤20
	NK-342	≥32	4/2	8/4	≤1	≤1	≤1	≤0.5	≤0.25	≤0.25	≤0.25	≥320
	NK-343	≥32	4/2	8/4	≤1	≤1	≤1	≤0.5	≤0.25	≤0.25	2	≤20
	NK-344	≥32	4/2	8/4	≤1	≤1	≤1	≤0.5	≤0.25	≤0.25	2	≤20
	NK-346	≥32	4/2	≤4/4	≤1	≤1	≤1	≤0.5	≤0.25	≤0.25	≤0.25	≥320
	NS-151	≤2	2/1	≤4/4	≤1	≤1	≤1	≤0.5	≤0.25	≤0.25	0.5	≥320
	NS-152*	≥32	≥32/16	≤4/4	≥16	4	≤1	≤0.5	0.5	≤0.25	≤0.25	≥320
	NS-153	≥32	4/2	≤4/4	≤1	≤1	≤1	≤0.5	≤0.25	≤0.25	≤0.25	≤20
	NS-154*	≥32	≥32/16	≤4/4	≥16	4	≤1	≤0.5	≤0.25	≤0.25	≤0.25	≥320
	NS-155	≤2	2/1	≤4/4	≤1	≤1	≤1	≤0.5	≤0.25	≤0.25	≤0.25	≥320
	NS-156*	≥32	≥32/16	≤4/4	≥16	4	≤1	≤0.5	1	≤0.25	≤0.25	≥320
	NS-157*	≥32	≥32/16	≤4/4	≥16	4	≤1	≤0.5	0.5	≤0.25	≤0.25	≥320
	NS-158*	≥32	≥32/16	≤4/4	≥16	4	≤1	≤0.5	≤0.25	≤0.25	≤0.25	≥320
	NS-161	≥32	4/2	≤4/4	≤1	≤1	≤1	≤0.5	≤0.25	≤0.25	≤0.25	≤20
	NS-162*	4	≥32/16	≤4/4	≤1	≤1	≤1	≤0.5	2	≤0.25	1	≥320
	NS-164*	≥32	≥32/16	≤4/4	≥16	4	≤1	≤0.5	≤0.25	≤0.25	≤0.25	≥320
F	NS-165*	≥32	≥32/16	≤4/4	≥16	4	≤1	≤0.5	0.5	≤0.25	≤0.25	≥320
	NS-170	≥32	4/2	≤4/4	≤1	≤1	≤1	≤0.5	≤0.25	≤0.25	≤0.25	≤20
	NS-172*	≥32	≥32/16	≤4/4	≥16	4	≤1	≤0.5	≤0.25	≤0.25	≤0.25	≥320
	NS-173	≥32	4/2	≤4/4	≤1	≤1	≤1	≤0.5	≤0.25	≤0.25	≤0.25	≤20
	NS-174	≤2	2/1	≤4/4	≤1	≤1	≤1	≤0.5	≤0.25	≤0.25	0.5	≥320
	NS-175	8	≥32/16	≤4/4	≤1	≤1	≤1	≤0.5	2	≤0.25	1	≤20
	NS-176*	≥32	≥32/16	≤4/4	≤1	≤1	≤1	≤0.5	2	≤0.25	2	≤20
	NS-177*	≥32	≥32/16	≤4/4	≥16	4	≤1	≤0.5	≤0.25	≤0.25	≤0.25	≥320
	NS-178	≥32	4/2	≤4/4	≤1	≤1	≤1	≤0.5	≤0.25	≤0.25	≤0.25	≤20
	NS-179	≥32	4/2	≤4/4	≤1	≤1	≤1	≤0.5	≤0.25	≤0.25	≤0.25	≤20
	NS-180*	≥32	≥32/16	≤4/4	≥16	4	≤1	≤0.5	0.5	≤0.25	≤0.25	≥320

	NS-182*	≥32	≥32/16	≤4/4	≥16	4	≤1	≤0.5	≤0.25	≤0.25	≤0.25	≥320
	NS-184	≤2	2/1	≤4/4	≤1	≤1	≤1	≤0.5	≤0.25	≤0.25	≤0.25	60
	NS-185*	≥32	≥32/16	≤4/4	≥16	4	≤1	≤0.5	≤0.25	≤0.25	≤0.25	≥320
	NS-187	≤2	2/1	≤4/4	≤1	≤1	≤1	≤0.5	≤0.25	≤0.25	≤0.25	60
	NY-039	≥32	4/2	≤4/4	≤1	≤1	≤1	≤0.5	≤0.25	≤0.25	≤0.25	≥320
	NY-040	≥32	4/2	≤4/4	≤1	≤1	≤1	≤0.5	≤0.25	≤0.25	0.5	≥320
	NY-041	≥32	4/2	≤4/4	≤1	≤1	≤1	≤0.5	≤0.25	≤0.25	≤0.25	≤20
	NY-042	≥32	4/2	≤4/4	≤1	≤1	≤1	≤0.5	≤0.25	≤0.25	≤0.25	≤20
	NY-043	≥32	4/2	≤4/4	≤1	≤1	≤1	≤0.5	≤0.25	≤0.25	≤0.25	≤20
	NY-045	≥32	4/2	≤4/4	≤1	≤1	≤1	≤0.5	≤0.25	≤0.25	≤0.25	≥320
	NY-046	≥32	4/2	≤4/4	≤1	≤1	≤1	≤0.5	≤0.25	≤0.25	≤0.25	≥320
	NY-048	≤2	2/1	≤4/4	≤1	≤1	≤1	≤0.5	≤0.25	≤0.25	≤0.25	≤20
	NY-049	≥32	4/2	≤4/4	≤1	≤1	≤1	≤0.5	≤0.25	≤0.25	0.5	≥320
	NY-050	≥32	4/2	≤4/4	≤1	≤1	≤1	≤0.5	≤0.25	≤0.25	0.5	≥320
	NY-051	≥32	4/2	≤4/4	≤1	≤1	≤1	≤0.5	≤0.25	≤0.25	≤0.25	≥320
	NY-052	≥32	4/2	≤4/4	≤1	≤1	≤1	≤0.5	≤0.25	≤0.25	≤0.25	≥320
	NY-053	≥32	4/2	≤4/4	≤1	≤1	≤1	≤0.5	≤0.25	≤0.25	≤0.25	≥320
	NY-054	≥32	4/2	≤4/4	≤1	≤1	≤1	≤0.5	≤0.25	≤0.25	0.5	≥320
	NY-056	≥32	4/2	≤4/4	≤1	≤1	≤1	≤0.5	≤0.25	≤0.25	0.5	≥320
	NY-057	≥32	4/2	≤4/4	≤1	≤1	≤1	≤0.5	≤0.25	≤0.25	1	≥320
	NY-059	≥32	4/2	≤4/4	≤1	≤1	≤1	≤0.5	≤0.25	≤0.25	0.5	≥320
	NY-065	≤2	2/1	≤4/4	≤1	≤1	≤1	≤0.5	≤0.25	≤0.25	≤0.25	≤20
	NY-095	≥32	4/2	≤4/4	≤1	≤1	≤1	≤0.5	≤0.25	≤0.25	≤0.25	≥320
	NY-097	≥32	8/4	≤4/4	≤1	≤1	≤1	≤0.5	≤0.25	≤0.25	≤0.25	≥320
	NY-209	≥32	4/2	≤4/4	≤1	≤1	≤1	≤0.5	≤0.25	≤0.25	≤0.25	≥320
	NY-210	≤2	2/1	≤4/4	≤1	≤1	≤1	≤0.5	≤0.25	≤0.25	≤0.25	≤20
	NY-212	≥32	4/2	≤4/4	≤1	≤1	≤1	≤0.5	≤0.25	≤0.25	≤0.25	≥320
G	NY-213*	≥32	8/4	8/4	≤1	≤1	≤1	≤0.5	≤0.25	≤0.25	4	60
	NY-214	≥32	4/2	≤4/4	≤1	≤1	≤1	≤0.5	≤0.25	≤0.25	0.5	≥320
	NY-215	≥32	4/2	≤4/4	≤1	≤1	≤1	≤0.5	≤0.25	≤0.25	0.5	≥320
	NY-216	≥32	4/2	≤4/4	≤1	≤1	≤1	≤0.5	≤0.25	≤0.25	1	≥320
	NY-217	≥32	4/2	≤4/4	≤1	≤1	≤1	≤0.5	≤0.25	≤0.25	0.5	≥320

NY-219	≥32	4/2	≤4/4	≤1	≤1	≤1	≤0.5	≤0.25	≤0.25	0.5	≥320
NY-220	≥32	4/2	≤4/4	≤1	≤1	≤1	≤0.5	≤0.25	≤0.25	0.5	≥320
NY-221	≥32	4/2	≤4/4	≤1	≤1	≤1	≤0.5	≤0.25	≤0.25	0.5	≥320
NY-222	≥32	4/2	≤4/4	≤1	≤1	≤1	≤0.5	≤0.25	≤0.25	0.5	≥320
NY-223	≥32	4/2	≤4/4	≤1	≤1	≤1	≤0.5	≤0.25	≤0.25	0.5	≥320
NY-319	≥32	4/2	≤4/4	≤1	≤1	≤1	≤0.5	≤0.25	≤0.25	≤0.25	≥320
NY-320	≥32	4/2	≤4/4	≤1	≤1	≤1	≤0.5	≤0.25	≤0.25	≤0.25	≥320
NY-321	≥32	4/2	≤4/4	≤1	≤1	≤1	≤0.5	≤0.25	≤0.25	0.5	≥320
NY-322	≤2	2/1	≤4/4	≤1	≤1	≤1	≤0.5	≤0.25	≤0.25	≤0.25	≤20
NY-323	≥32	4/2	≤4/4	≤1	≤1	≤1	≤0.5	≤0.25	≤0.25	≤0.25	≥320
NY-324	≥32	4/2	≤4/4	≤1	≤1	≤1	≤0.5	≤0.25	≤0.25	≤0.25	≥320
NY-325	≥32	4/2	≤4/4	≤1	≤1	≤1	≤0.5	≤0.25	≤0.25	≤0.25	≥320
NY-326*	≥32	≥32/16	32/4	8	≤1	≤1	≤0.5	≤0.25	≤0.25	4	≤20
NY-327	≥32	4/2	≤4/4	≤1	≤1	≤1	≤0.5	≤0.25	≤0.25	≤0.25	≤20
NY-328	≥32	4/2	≤4/4	≤1	≤1	≤1	≤0.5	≤0.25	≤0.25	≤0.25	≥320
NY-329	≥32	4/2	≤4/4	≤1	≤1	≤1	≤0.5	≤0.25	≤0.25	≤0.25	≥320
NY-334	≥32	4/2	≤4/4	≤1	≤1	≤1	≤0.5	≤0.25	≤0.25	≤0.25	≥320
NY-335	≥32	4/2	≤4/4	≤1	≤1	≤1	≤0.5	≤0.25	≤0.25	≤0.25	≥320
NY-337	≥32	4/2	≤4/4	≤1	≤1	≤1	≤0.5	≤0.25	≤0.25	≤0.25	≥320
NY-338*	≥32	4/2	8/4	≤1	≤1	≤1	≤0.5	≤0.25	≤0.25	2	60
NY-340	≥32	4/2	8/4	≤1	≤1	≤1	≤0.5	≤0.25	≤0.25	2	≤20
NY-345	≤2	2/1	≤4/4	≤1	≤1	≤1	≤0.5	≤0.25	≤0.25	≤0.25	≥320

*Multidrug-resistant
