# Supplementary material for: Antimicrobial Susceptibility and Frequency of bla and qnr Genes in Salmonella enterica Isolated from Slaughtered Pigs
Source: Antibiotics (Basel). 2021 Nov 24;10(12):1442. doi: 10.3390/antibiotics10121442 (PMC8698178; doi:10.3390/antibiotics10121442)
Supplement: Supplementary file 1 [file antibiotics-10-01442-s001.zip › TableS2.pdf]

Table S2. Distribution of *S. enterica* into serogroups and H1/Sdf I typing.

Abattoir	Isolate	Serogroup	H1/Sdf I
	AC-003	O:7	g
	AC-004	O:3	r
	AC-005	O:7	Other
	AC-006	O:3	r
	AC-007	O:7	r
	AC-008	O:7	r
	AC-009	O:8	i
	AC-010	O:7	r
	AC-253	O:3	Other
	AC-254	O:4	Other
	AC-255	O:7	g
	AC-256	O:7	g
	AC-257	O:3	e,h
	AC-258	O:7	g
A	AC-259	O:7	i
	AC-260	O:7	g
	AC-261	O:7	g
	AC-262	O:7	g
	AC-263	O:7	g
	AC-264	O:7	i
	AC-265	O:7	r
	AC-267	O:7	r
	AC-268	O:7	i
	AC-270	O:7	g
	AC-272	O:7	i
	AC-273	O:7	i
	AC-274	O:7	i
	AC-275	O:7	g
	AC-276	O:7	g

B	AM-102	O:7	e,h
	AM-105	O:3	r
	AM-106	O:7	e,h
	AM-108	O:3	Other
	AM-109	O:7	i
	AM-112	O:7	g
	AM-113	O:7	g
	AM-114	O:7	g
	AM-115	O:3	r
	AM-116	O:3	e,h
	AM-118	O:4	e,h
	AM-119	O:3	r
	AM-120	O:4	i
	AM-121	O:8	Other
	AM-191	O:7	i
	AM-192	O:4	i
	AM-193	O:3	Other
	AM-247	O:3	e,h
	AM-248	O:3	e,h
	AM-249	O:3	e,h
	AM-250	O:3	e,h
	AM-251	O:3	e,h
	AM-252	Other	N/A
C	AT-098	O:3	e,h
	AT-099	O:3	e,h
D	AV-066	O:4	e,h
	AV-067	O:9	sdf
	AV-068	O:9	sdf
	AV-070	O:4	Other
	AV-072	O:3	e,h
	NK-011	O:4	i
	NK-012	O:4	r

	NK-013	Other	N/A
	NK-015	O:4	Other
	NK-016	O:3	r
	NK-017	O:3	r
	NK-018	O:3	e,h
	NK-019	O:9	sdf
	NK-021	O:4	i
	NK-022	O:4	i
	NK-023	O:3	Other
	NK-024	O:4	Other
	NK-025	O:4	Other
	NK-026	O:4	i
	NK-027	O:3	r
	NK-029	O:3	r
	NK-031	O:4	Other
	NK-032	O:4	i
	NK-033	O:3	r
	NK-035	O:4	i
	NK-036	O:4	i
E	NK-037	O:4	i
	NK-093	O:3	r
	NK-094	O:3	r
	NK-194	O:3	e,h
	NK-195	O:3	e,h
	NK-196	O:3	e,h
	NK-197	O:3	e,h
	NK-198	O:4	i
	NK-199	O:3	Other
	NK-200	O:3	e,h
	NK-201	O:4	i
	NK-202	O:4	i
	NK-203	O:4	i

	NK-204	O:4	i
	NK-205	O:4	i
	NK-206	O:4	i
	NK-208	O:3	Other
	NK-340	O:4	i
	NK-341	Other	N/A
	NK-342	O:3	e,h
	NK-343	O:4	i
	NK-344	O:4	i
	NK-346	O:7	g
	NS-151	O:3	e,h
	NS-152	O:3	e,h
	NS-153	O:3	e,h
	NS-154	O:3	e,h
	NS-155	O:3	e,h
	NS-156	O:3	e,h
	NS-157	O:3	e,h
	NS-158	O:3	e,h
	NS-161	O:3	e,h
	NS-162	Other	N/A
	NS-164	O:3	e,h
	NS-165	O:3	e,h
F	NS-170	O:3	e,h
	NS-172	O:3	e,h
	NS-173	O:3	e,h
	NS-174	O:3	e,h
	NS-175	Other	N/A
	NS-176	Other	N/A
	NS-177	O:3	e,h
	NS-178	O:3	e,h
	NS-179	O:3	e,h
	NS-180	O:3	e,h

	NS-182	O:4	i
	NS-184	Other	N/A
	NS-185	Other	N/A
	NS-187	O:3	Other
	NY-039	O:7	r
	NY-040	Other	N/A
	NY-041	O:3	r
	NY-042	O:3	r
	NY-043	O:3	r
	NY-045	O:4	Other
	NY-046	O:3	r
	NY-048	O:4	d
	NY-049	O:7	Other
	NY-050	O:7	Other
	NY-051	Other	N/A
	NY-052	O:7	g
	NY-053	O:3	r
	NY-054	O:7	g
	NY-056	O:7	Other
	NY-057	O:7	g
	NY-059	O:7	Other
	NY-065	O:7	i
	NY-095	O:7	g
	NY-097	O:7	Other
	NY-209	O:8	i
	NY-210	Other	N/A
	NY-212	O:3	e,h
	NY-213	O:4	i
G	NY-214	O:7	r
	NY-215	O:7	r
	NY-216	O:7	r
	NY-217	O:7	r

NY-219	O:4	Other
NY-220	O:7	g
NY-221	O:7	g
NY-222	O:7	g
NY-223	O:7	g
NY-319	O:3	e,h
NY-320	O:3	r
NY-321	O:7	g
NY-322	O:4	i
NY-323	O:3	r
NY-324	O:3	r
NY-325	O:3	r
NY-326	O:4	Other
NY-327	O:3	e,h
NY-328	O:4	i
NY-329	O:3	r
NY-334	O:7	g
NY-335	O:7	g
NY-337	O:7	g
NY-338	O:4	i
NY-345	O:3	r

N/A - Subtyping for unidentified serogroups were not attempted.
